# Estimating trauma prevalence from incomplete human skeletal remains

**DOI:** 10.1038/s41598-024-76231-1

**Published:** 2024-11-12

**Authors:** Judith Beier, Matteo Santon, Hannes Rathmann

**Affiliations:** 1 DFG Center for Advanced Studies ‘Words, Bones, Genes, Tools’, University of Tübingen, 72070 Tübingen, Germany; 2 Paleoanthropology, Department of Geosciences, Institute for Archaeological Sciences, University of Tübingen, 72070 Tübingen, Germany; 3 Ecology of Vision Group, School of Biological Sciences, University of Bristol, Bristol, BS8 1TQ UK; 4 Senckenberg Centre for Human Evolution and Palaeoenvironment, University of Tübingen, 72070 Tübingen, Germany

**Keywords:** Trauma prevalence, Crude frequencies, Generalized linear (mixed) models, Skeletal preservation, Simulations, Biological anthropology, Archaeology

## Abstract

Traumatic lesions on human skeletal remains are widely used for reconstructing past accidents or violent encounters and for comparing trauma prevalence across samples over time and space. However, uncertainties in trauma prevalence estimates increase proportionally with decreasing skeletal completeness, as once-present trauma might have gone missing. To account for this bias, samples are typically restricted to skeletal remains meeting a predefined minimum completeness threshold. However, the effect of this common practice on resulting estimates remains unexplored. Here, we test the performance of the conventional frequency approach, which considers only specimens with ≥ 75% completeness, against a recent alternative based on generalized linear models (GLMs), integrating specimen completeness as a covariate. Using a simulation framework grounded on empirical forensic, clinical, and archaeological data, we evaluated how closely frequency- and GLM-based estimates conform to the known trauma prevalence of once-complete cranial samples after introducing increasing levels of missing values. We show that GLM-based estimates were consistently more precise than frequencies across all levels of incompleteness and regardless of sample size. Unlike GLMs, frequencies increasingly produced incorrect relative patterns between samples and occasionally failed to produce estimates as incompleteness increased, particularly in smaller samples. Consequently, we generally recommend using GLMs and their extensions over frequencies, although neither approach is fully reliable when applied to largely incomplete samples.

## Introduction

Trauma analyses are an integral part of bioarchaeological investigations. Traumatic lesions result from accidents or violent encounters, and their examination represents a powerful tool to gain insights into past humans’ fates and behaviors. The level and patterns of trauma present in a population allow for reconstructions of group-specific social, cultural, and environmental life realities and their comparisons across time and space^1–10^.

Trauma is commonly recorded as a binary variable (present/absent) for each bone or individual. Trauma prevalence in a sample is typically given as a percentage, representing the proportion of individuals or skeletal parts with injuries, and compared between samples using statistical tests such as chi-squared or Fisher’s exact test. Such crude trauma frequencies (hereafter, CFs) are directly affected by skeletal preservation in both qualitative and quantitative means (hereafter, completeness), because once-present traumatic lesions might be missing from an incompletely preserved skeletal record^11–13^. Hence, uncertainties in trauma prevalence estimates increase proportionally with decreasing completeness of the remains and may result in false negative findings in incomplete samples where once-present trauma might have gone missing^11,12,14–16^. To account for this potential bias in estimates of trauma prevalence and their statistical analysis, samples are commonly restricted to skeletal remains in a pre-defined minimum completeness state, while neglecting less complete individuals or bones. However, the threshold at which completeness is deemed sufficient for inclusion varies among studies, and various thresholds have been employed, such as 25%^17^, 40%^18^, 50%^18–21^, 66,7%^22,23^, 80%^12,24^, 90%^25^, 100%^13^, or most commonly, 75%^11,12,14,15,19,24,26–31^. Other methodological differences exist, for example, in handling trauma observations; while some studies exclude trauma findings on remains smaller than the defined threshold^11,13,15,26,28,29,31^, others include them to maintain a wider range of analyzable variation^12,18,24,25^.

Employing CFs along with completeness thresholds enhances data quality and comparability between samples but, at the same time, severely reduces sample sizes if samples are overall poorly preserved. This issue especially applies to the human fossil record, which comprises large portions of partial remains. The use of alternative approaches, such as generalized linear models (hereafter, GLMs), to estimate and compare trauma rates within and between samples can help overcome the trade-off between sample sizes and comparability that occurs in the CF approach when dealing with incompletely preserved samples. GLMs are an extension of linear models, which describe the relationship between one response variable as a function of one or multiple predictors^32^. However, instead of assuming a Gaussian distribution family, GLMs are implemented with other distributions to analyse skewed, count, proportional, or, in our case, binary response variables such as trauma scores^32–35^. Moreover, GLMs allow for the simultaneous assessment of multiple predictor variables and their interactions, such as sex, age at death, or archaeological period. Importantly, this approach allows the inclusion of all available skeletal fragments for analysis, regardless of their size and without the need for a priori exclusion, achieved by assigning a completeness score to each fragment and incorporating completeness as a predictor. By adopting such a modeling approach, Beier et al.^36,37^ investigated cranial trauma rates in highly fragmented Late Pleistocene human fossils from Eurasia, including even the smallest skeletal elements preserved, while factoring in variation in trauma prevalence owing to differential completeness. Given the advantages of maximizing sample sizes and evaluating multiple predictor variables simultaneously, they recommended utilizing GLMs and related techniques instead of CFs to estimate trauma prevalence in samples of fossilized or otherwise incomplete skeletal remains. However, this recommendation has not been analytically corroborated, prompting them to advise an evaluation of the performance of the two approaches in a designated study in the future.

In this study, we employ a simulation framework based on empirical forensic, clinical, and archaeological data to explicitly test how closely CF- and GLM-based trauma prevalence estimates align with the known prevalence of once-complete skeletal samples after introducing increasing amounts of missing values. Our focus is solely on exploring the performance of these two common approaches in capturing the true trauma rate in incomplete samples. We do not address the statistical comparison between samples, which may also be part of bioarchaeological trauma investigations, such as when testing hypotheses about inter- and intra-sample trauma rates, including differences between sexes, sites, or age cohorts.

## Methods

**Fig. 1:**
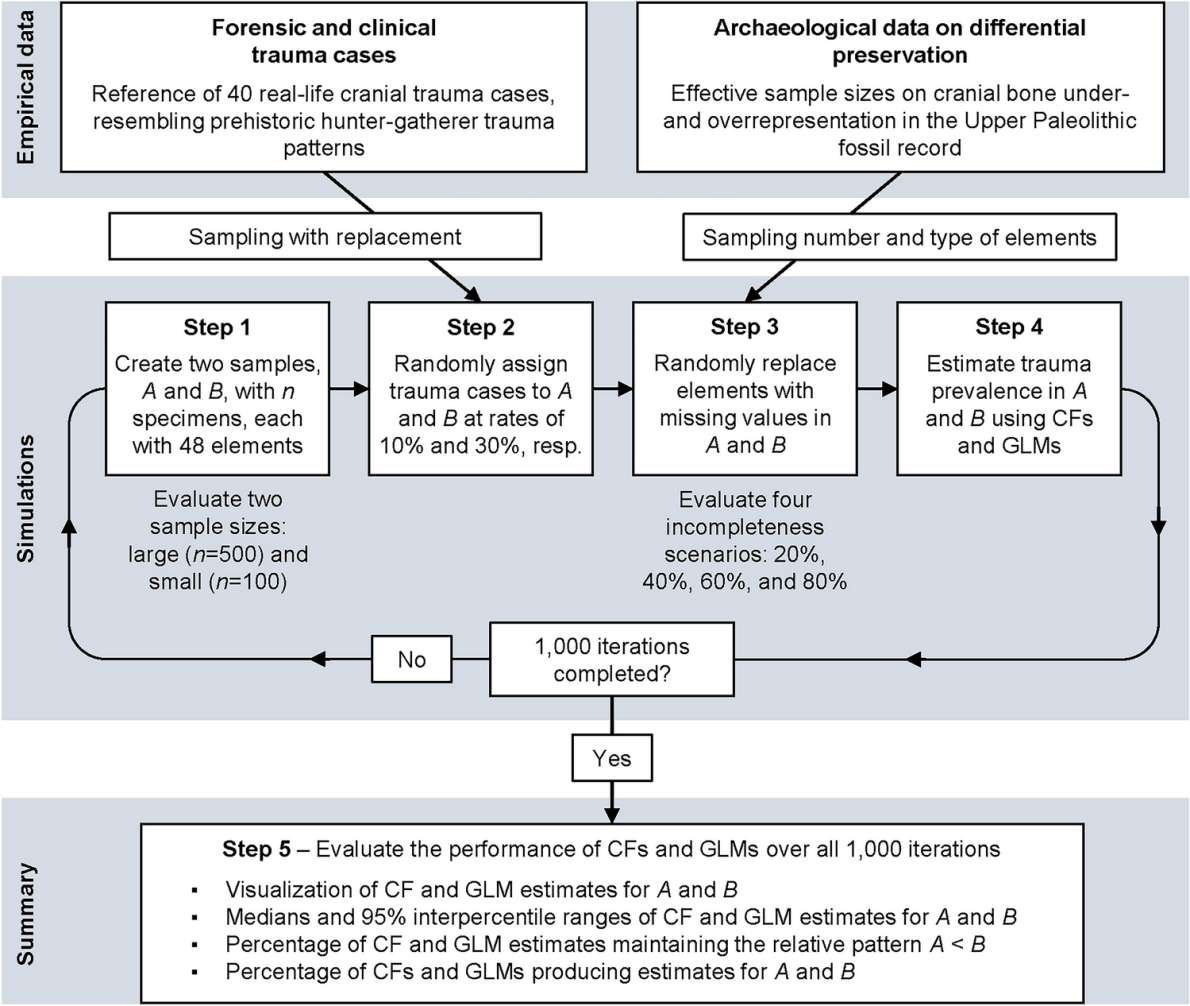
Workflow summarizing all key stages of our simulation framework. In each simulation run, we first created an artificial dataset with two samples, A and B, each comprising *n* specimens. Each specimen was represented by a vector of 48 cranial elements, serving both as a scheme for recording trauma and for allocating missing values. We evaluated large (*n* = 500) and small (*n* = 100) initial sample sizes. Second, we defined 10% of the specimens in sample A to be affected by trauma, and 30% in sample B. Trauma cases were randomly sampled from a pool of 40 real-life forensic and clinical trauma cases, approximating injury scenarios and patterns that are realistic for prehistoric hunter-gatherer populations. Third, missing values were randomly added to the specimens in samples A and B, allowing some specimens to have more missing data than others, mimicking variation in preservation in the archaeological skeletal record. Drawing on empirical data from the Upper Paleolithic fossil record^37^, we gave cranial elements typically underrepresented in the skeletal record a higher likelihood of being assigned missing values, while those commonly well-represented were less likely to be removed. We evaluated four incompleteness scenarios with increasing levels of data missingness (on average, 20%, 40%, 60%, and 80% incomplete). Fourth, the incomplete data was used to generate CF- and GLM-based trauma prevalence estimates for samples A and B. In the CF approach, trauma frequency was estimated only over specimens with ≥ 75% completeness, while excluding all less complete remains with and without trauma. In the GLM approach, we generated model-derived point estimates of trauma prevalence prediction, using specimen completeness as a continuous covariate. For each scenario, we ran 1000 simulation iterations. Fifth, we quantified from the resulting distributions of trauma prevalence estimates: (1) the medians to assess the two approaches’ overall accuracy in relation to the benchmarks; (2) the 95% interpercentile ranges to assess the approaches’ precision; (3) the percentage of CF and GLM estimates that maintained the correct relative prevalence pattern between samples A and B; and (4) the percentage of how often the two approaches were able to produce prevalence estimates for either sample.

Figure 1 summarizes all key stages of our simulation framework. In each simulation run, we first created an artificial dataset with two samples, A and B, each comprising *n* specimens. For simplicity, and in line with our previous research, our simulation approach focuses solely on the skull. Each specimen was represented by a vector of cranial elements, which served both as a scheme for recording trauma and for allocating missing values across the skull. We subdivided the skull into 14 major bones, namely, the frontal and occipital bones, as well as the left and right elements of the parietal, temporal, maxilla, mandible, zygomatic and nasal bones. Each cranial bone was further divided into four roughly equal-sized regions, oriented, for example, on anatomical structures or midlines. Due to their small size, the left and right nasal and zygomatic bones were divided into just two regions. The partitioning resulted in a total of 48 cranial elements per skull. While other procedures for assessing skeletal completeness exist^14,38–41^, we followed the cranial bone partitioning and trauma recording protocol previously employed by one of us^36,37^, allowing us to incorporate existing empirical data on cranial completeness into our simulations (see below). We performed our simulations once with large initial (i.e., before introducing missing values) sample sizes for A and B (*n* = 500) and once with small initial sample sizes (*n* = 100).

In the second step, we defined 10% of the specimens in sample A to be affected by traumatic injuries, and 30% in sample B. These arbitrary numbers reflect an average cranial trauma rate on one hand, and a slightly elevated rate on the other. For example, trauma rates around 10% are commonly found in Neolithic cranial samples^42^, whereas highly traumatized samples such as those resulting from violent massacres show a cranial trauma prevalence of up to 50%, or even more^9,42,43^. Trauma on any of the 48 cranial elements was recorded as either absent (i.e., ‘0’) or present (i.e., ‘1’). Because traumatic fractures are not arbitrarily distributed across the skull but follow distinct biomechanical breakage patterns^44–48^, we used real-life forensic and clinical trauma cases as a reference. We compiled a set of 40 trauma cases from publications and online resources, selecting only injury scenarios that are realistic for prehistoric hunter-gatherer populations. We focused on blunt force trauma resulting from interpersonal violence (without modern-day weapons or tools), falls from a height, animal encounters, as well as projectile trauma from arrow shots. We excluded sharp force trauma, high-velocity trauma such as motor vehicle accidents or plane crashes, explosives, and gunshot trauma, as these represent injury causes with distinct trauma patterns that were not present in the deep past, such as during the Paleolithic. Being composed of both forensic and clinical cases, the severity of trauma in the selected cases ranges from minor (with only a few ‘1s’ distributed over the 48 cranial elements) to extensive and fatal (with multiple ‘1s’ distributed over the 48 cranial elements). Details on the fractures, injury situations, and associated references are provided in Table 1. Further information about the proportion of individual elements affected by trauma within the pool of real-life trauma cases is provided in the Supplementary Information Figure S1. From the reference pool of 40 trauma cases, we randomly assigned cases to samples A and B at rates of 10% and 30%, respectively, using sampling with replacement, meaning each case always had the same chance of being selected in every draw. Consequently, some trauma cases could appear once or multiple times in either sample, while others might not be selected at all. This sampling procedure ensures that our simulations are not systematically biased towards a possible over- or underrepresentation of minor or extensive trauma cases in either sample. This is crucial because extensive trauma cases involving multiple skeletal elements of a skull are more likely to persist in a sample when skeletal preservation decreases.

**Table 1:** Clinical and forensic trauma cases used as a reference.

Trauma category	Age	Sex	Situation	Trauma type	Nr. of injured elements	Case ID	References
Sports accident	35	Male	Headbutt from opposition player in football game	Depressed fracture on right frontal	1	97910	^49^
Sports accident	25	–	Football tackle	Fractures on the left mandibular body and the right mandibular subcondylar area	3	89829	^50^
Animal encounter	Adult	Male	Kicked in the head by horse	Depressed fracture on right parietal	4	–	^51^
Animal encounter	28	Male	Tamer attacked by three tigers during evening show, bite to the head	Penetrating fractures from tigers’ canines and associated linear fractures on left parietal, temporal and occipital	6	–	^52^
Animal encounter	35	Male	Attacked by bear in the wild, hit with paw	Multiple fractures of the right zygomatic arch, zygoma, and maxilla	6	Case 1	^53^
Animal encounter	18	Female	Attacked by bear in the wild from behind, first hit with paw	Comminuted fractures of the left zygoma, zygomatic arch, and maxilla	5	Case 2	^53^
Animal encounter	16	Male	Attacked by tiger in the wild, single hit with the paw	Multiple fractures of the left mandibula (posterior body and subcondylar area), left zygomatic arch, left zygoma, and left and right maxillae	9	Case 3	^53^
Animal encounter	50	Female	Kicked and trampled by a horse	Comminuted fractures of the right cranioafacial region involving the frontal, maxilla, nasal bone, zygoma, and zygomatic arch	9	44849	^54^
Fall from a height	80	Male	Fell from a roof	Occipital condyle fracture	1	87674	^55^
Fall from a height	50	Male	Fall	Fractures of the right mandibular body and subcondylar area	2	56134	^56^
Fall from a height	Young adult	Female	Jumped from 7^th^ floor of a building (approx. 21 m), landed on lower balcony	Extensive comminuted fractures across the cranial vault, face, and mandible	34	FT-1	^57^
Fall from a height	Young adult	Female	Jumped from a bridge (approx. 58 m), landed in a river	Fractures of the left mandibular body and the right mandibular angle	3	FT-2	^57^
Fall from a height	Young adult	Male	Jumped from a bridge (approx. 58 m), landed in a river	Linear fracture of the left maxilla	1	FT-3	^57^
Fall from a height	Middle aged adult	Female	Jumped off a cliff (approx. 45 m), landed on sand, possible impacts with the cliff edge during fall	Extensive comminuted fractures across the face, mandible, and cranial vault, particularly on the left side	19	FT-4	^57^
Fall from a height	Elderly adult	Male	Fell from the roof of a house (approx. 3 m), through a plastic covering, landed on concrete floor	Linear fractures of the left and right temporal and parietal bones and the occipital	8	FT-12	^57^
Fall from a height	Adolescent	Male	Fell from a bridge (approx. 15 m) onto a footpath	Linear fractures of the right temporal and parietal bones and the occipital	4	FT-14	^57^
Fall from a height	Elderly adult	Male	Jumped headfirst from ladder, fell approx. 2 m, landed on concrete floor	Linear fractures of the left frontal and maxilla	4	FT-8	^57^
Fall from a height	Elderly adult	Male	Suffered from natural heart disease event causing him to fall approx. 3 m from a building framework without attempt to break the fall	Multiple fractures across the frontal, occipital, left parietal and maxilla	8	FT-16	^57^
Fall from a height	30	Male	Fell headlong from 2 m	Linear fracture of the frontal and right parietal	4	23979	^58^
Miscellaneous clinical	15	Female	Facial trauma 8 years back, healed	Complete bone ankylosis of the right temporomandibular joint	3	88120	^59^
Miscellaneous clinical	25	Male	Nasal bone fracture	Left and right nasal bone fracture	2	91256	^60^
Miscellaneous clinical	30	Male	Blunt frontal trauma	Depressed frontal bone fracture involving the frontal sinus	2	90655	^61^
Miscellaneous clinical	40	Male	Nasal bone fracture	Left and right nasal bone fracture	2	68197	^62^
Miscellaneous clinical	Adult	–	Depressed skull fracture	Depressed fracture of left parietal	1	13084	^63^
Projectile/arrow shot	25	Male	Arrow injury to the face	Penetrating fracture through the left mandibular ramus	1	73267	^64^
Projectile/arrow shot	35	Male	Arrow shot through the forehead	Penetrating fracture through the frontal bone at the right frontal sinus	1	72101	^65^
Violence	Adolescent	Male	Punch to head with fist causing fall to the ground	Linear fracture of the right parietal	2	BFT-2	^66^
Violence	Young adult	Male	Punch to head with fist causing fall backward onto concrete pavement	Fractures of the occipital bone at the foramen magnum and the left petrous bone	4	BFT-3	^66^
Violence	Middle aged adult	Male	Punched with fist, kicked with feet (no shoes), struck with statue and chair	Multiple fractures of the face involving the frontal and nasal bones, zygomas, zygomatic arches, maxillae, and mandible	19	BFT-8	^66^
Violence	Middle aged adult	Female	Assault: blunt-force head injury and compressive neck injury inflicted with hands	Fractures of the left zygoma, zygomatic arch and maxilla	5	BFT-10	^66^
Violence	Elderly adult	Male	Assault: fell, dragged, and disposed in creek, beaten with tree branch	Multiple fractures of the left frontal and temporal bones, nasal bones, mandible, and maxillae	19	BFT-15	^66^
Violence	45	Male	Assault	Subcondylar mandibular fracture on the right side	1	155152	^67^
Violence	20	Male	Punched in the jaw	Fracture of the left mandibular body	1	82151	^68^
Violence	20	Male	Alleged assault	Fracture of the left mandibular body	2	57289	^69^
Violence	25	Male	Punched in the jaw	Fractures of the right mandibular body and the left angle	4	54433	^70^
Violence	25	Male	Altercation, punched in the face	Fracture of the left zygomatic arch	2	48597	^71^
Violence	20	Male	Multiple punches to the face	Fracture of the right zygomatic arch	1	47820	^72^
Violence	16	Male	Hit with a stick	Depressed fracture of the right anterior maxillary sinus wall	1	97910	^73^
Violence	15	Male	Fight at school	Fractures of left and right nasal bones and left maxillar frontal process	4	89829	^74^
Violence	Young adult	Female	Assault	Fractures of the right temporal and parietal	3	-	^75^

Individual fracture patterns were recorded across the 48 cranial elements from published images and online CT representations. Diastatic fractures were disregarded due to their limited recognizability in a fragmentary archaeological skeletal record. Column “Nr. of injured elements” indicates how many of the 48 cranial elements were affected by trauma. Raw data are given in the R code (Supplementary Information Code S1).

In the third step, we introduced missing values per specimen in samples A and B by deleting trauma absence/presence scores recorded on any of the 48 cranial elements (i.e., replacing ‘0’ or ‘1’ for a given cranial element with a missing value). This procedure may remove portions of a previously larger fracture, i.e., when one or more elements affected by a fracture extending over multiple elements are removed, but not all. This is intentional, as larger fractures may be partially preserved in the archaeological record if crania are incompletely preserved. We evaluated four incompleteness scenarios with increasing levels of overall data missingness: on average, 20%, 40%, 60%, and 80% incomplete. The frequency of missing values introduced per specimen in each of the four incompleteness scenarios was not fixed. Instead, it was randomly picked from a normal distribution with a mean corresponding to either 20%, 40%, 60%, or 80%, and a standard deviation of 20. If the sampled value fell below 0%, it was set to 0%. Similarly, if the sampled value exceeded 100%, it was capped at 100%. For instance, in the 40% incomplete scenario, most specimens have around 40% missing values, with some having more, perhaps up to 100%, while others having less, perhaps 0%. This procedure allows for variability, ensuring that, in each overall incompleteness scenario, all specimens had similar but slightly different missing data amounts, mimicking preservation-related variation in the archaeological skeletal record.

The randomly selected number of missing values per specimen was then allocated to the 48 cranial elements again in a random manner. Previous research^37,76^ has shown that some cranial elements, such as the mandible, frontal, and parietal bones, are more likely represented in the archaeological record than others, such as the nasal bones and zygoma, owing to various taphonomic factors, including differential bone structure properties and decomposition or animal and human activity^39,76^. To account for this variability in skeletal representation, we incorporated probability weights in the random allocation of missing values to the 48 cranial elements per specimen. Weights were obtained from published skeletal element completeness counts of an Upper Paleolithic cranial sample (~ 40,000–10,000 years before present) from Beier et al.^37^ (see their Table 1; see also Beier^77^ for raw data). From this data, we calculated ‘effective sample sizes’ for each of the 14 cranial bones by adding up fractional elements to ‘complete’ bones (i.e., the number of elements preserved per completeness category multiplied by their completeness scores, i.e., 0.25, 0.5, 0.75, 1). This approach has been described by Walker^78^ to quantify how much a bone fragment contributes to the total sample, though for individuals instead of single cranial bones as used here. The obtained effective sample sizes used for weighing are given in the code (Supplementary Information Code S1) and are illustrated in Supplementary Information Figure S1. The weighting procedure ensures that commonly poorly represented cranial bones, such as mid-facial bones^37^ (Fig. S1), have a higher likelihood of being allocated a missing value for one or several of their elements, while commonly well-represented cranial bones and their elements, such as vault bones and the mandible^37^ (Fig. S1), are less likely to be removed.

In the fourth step, we recorded for each specimen: (1) the completeness, defined as the percentage of remaining cranial elements out of 48 (ranging from 0 to 100% complete), and (2) whether any of the remaining elements exhibit a trauma or not (recorded as either ‘0’ or ‘1’). This summary data per specimen was then used to estimate trauma prevalence with the two different approaches: CFs and GLMs. In the CF approach, the trauma frequency in samples A and B was estimated only over specimens with ≥ 75% completeness, while excluding all less complete remains with and without trauma, following common practice in bioarchaeological trauma studies^11,15,26,28,29,31^. In the GLM approach, the response variable (absence or presence of trauma) was modelled using a binomial distribution (link = log). We used the categorial predictor ‘sample’ (A or B) and the continuous covariate ‘completeness’ as fixed predictors in the models. We implemented the GLMs with the *glmmTMB* R-package^79^, following a custom-written guided linear modelling R-routine^35^. Model assessment involved the inspection of the distribution of randomized quantile residuals^80^ and posterior predictive checks to assess overall model fit^35^. From the posterior distributions of fitted values obtained from 10,000 sets of model parameters, we predicted trauma prevalence for samples A and B for the 75% to 100% completeness range (to ensure comparability between GLM- and CF-based estimates) and averaged those estimates to arrive at point estimates of trauma prevalence prediction.

For both large (*n* = 500) and small (*n* = 100) initial sample sizes, and for each of the four incompleteness scenarios (on average, 20%, 40%, 60%, and 80% missing data), we ran 1000 simulation iterations. In the fifth and final step, we evaluated the performance of CFs and GLMs over all 1000 iterations and (1) visually displayed the shape of the resulting distributions of trauma prevalence estimates using sina plots and error bars; (2) quantified the medians to assess the overall accuracy of CF- and GLM-based trauma prevalence estimates in relation to the benchmarks; (3) estimated the 95% interpercentile ranges to assess their precision; (4) calculated the percentage with which each approach maintained the correct relative prevalence pattern between samples A and B; and (5) quantified the percentage of how often each approach was able to produce prevalence estimates for either sample.

All analyses were performed in the programming language R^81^. Graphics were created using the package ggplot2^82^.

## Results

### Initial sample sizes of 500 specimens

**Fig. 2:**
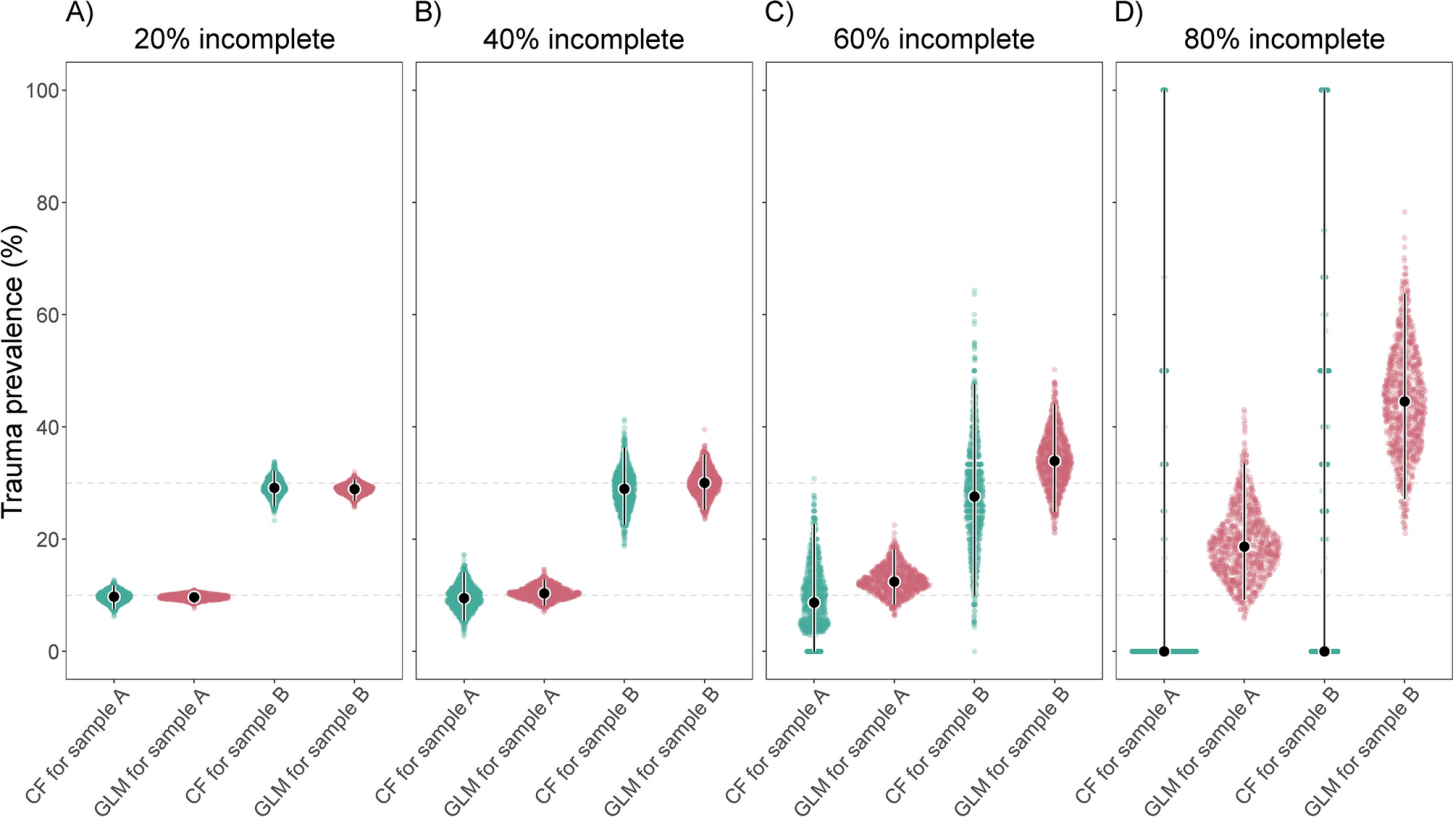
Sina plots showing the performance of CFs and GLMs in estimating cranial trauma prevalence for samples A and B (initially each *n* = 500) with increasing levels of data missingness, assessed with 1000 simulated datasets each: (**A**) 20% missing data; (**B**) 40% missing data; (**C**) 60% missing data; (**D**) 80% missing data. Color-coding denotes CF- (green) and GLM-derived (red) estimates. Dotted horizontal lines illustrate the prevalence benchmarks for samples A and B (10% and 30%, respectively). Error bars are superimposed on the distributions to display medians (dots; indicating accuracy in relation to the benchmarks) and 95% interpercentile ranges (bars; indicating precision).

Figure 2 displays the performance of CFs and GLMs in estimating cranial trauma prevalence for samples A and B as the level of missing data increases, with each sample consisting of 500 specimens initially. The corresponding values are presented in Table 2a. When the amount of missing data is low to moderate (20% and 40% incomplete), CFs and GLMs produced similarly accurate estimates for both samples (i.e., the medians of the spread of both CF and GLM estimates were similarly close to the benchmarks of samples A and B). However, with high to excessive amounts of missing data (60% and 80% incomplete), CFs tended to increasingly underestimate the benchmarks for both samples, while GLMs tended to overestimate them. Notably, GLM estimates were consistently more precise than CF estimates across all levels of incompleteness for both samples (i.e., the 95% interpercentile ranges of the spread of GLM estimates were narrower compared to those of CF estimates). This difference in precision becomes most pronounced in the 80% missing data scenario, where CF-based prevalence estimates span the entire range from 0 to 100% in both samples A and B. Moreover, note that CF-based estimates become increasingly skewed towards 0% as the amount of missing data increases, starting at 60% incomplete, particularly for sample A. For instance, in the 80% missing data scenario, CFs yielded a prevalence of 0% in 82.8% of simulations for sample A and in 53% for sample B, although the simulated raw data with missing values never exhibited a prevalence of 0%. In contrast, GLMs always produced prevalence estimates > 0%, in compliance with the simulated raw data.

**Table 2:** Summary statistics on the performance of CFs and GLMs in estimating cranial trauma prevalence for samples A and B (benchmarks: 10% and 30%, respectively) with increasing levels of data missingness (on average 20%, 40%, 60%, and 80% incomplete), each of the four incompleteness scenarios assessed with 1000 simulated datasets. Initial sample sizes for samples A and B are 500.

(a) Median (and 95% interpercentile range) of trauma prevalence estimates				
Scenario	CF		GLM	
	Sample A	Sample B	Sample A	Sample B
20%	9.748 (7.616–11.726)	29.132 (25.937–32.144)	9.656 (8.659–10.517)	28.916 (26.896–30.690)
40%	9.483 (5.453–14.050)	28.947 (22.608–36.363)	10.343 (8.213–12.749)	30.020 (25.269–35.059)
60%	8.696 (0.000–22.736)	27.586 (10.000–47.624)	12.426 (8.392–18.197)	33.925 (24.881–43.979)
80%	0.000 (0.000–100.000)	0.000 (0.000–100.000)	18.665 (9.261–33.494)	44.518 (27.173–63.727)

(b) Percentage of maintaining the correct relative prevalence pattern between samples A and B				
Scenario	CF		GLM	
20%	100%		100%	
40%	100%		100%	
60%	94.7%		100%	
80%	29%		100%	

(c) Percentage of producing a trauma prevalence estimate for samples A and B				
Scenario	CF		GLM	
	Sample A	Sample B	Sample A	Sample B
20%	100%	100%	100%	100%
40%	100%	100%	100%	100%
60%	100%	100%	100%	100%
80%	81%	83.2%	100%	100%

GLMs consistently maintained the correct relative trauma prevalence pattern between samples A and B (with A < B) across all levels of incompleteness (Table 2b). In contrast, CFs only maintained the correct relative trauma prevalence pattern when the amount of missing data was low to moderate (20% and 40% incomplete). With high to excessive amounts of missing data (60% and 80% incomplete), CFs maintained the correct relative pattern in only 94.7% and 29% of simulations, respectively, while in the remaining cases they yielded an incorrect, reversed pattern (with A > B). An examination of the simulated raw datasets revealed that the A < B pattern was always maintained, despite the increasing amounts of missing values. While GLM estimates consistently reproduced this pattern given the raw data at hand, the CF estimates failed increasingly to do so, likely due to the excessive exclusion of specimens that were less than 75% complete.

GLMs always produced trauma prevalence estimates for samples A and B across all levels of incompleteness (Table 2c). CFs only consistently produced trauma prevalence estimates when the amount of missing data ranged from low to high (20%, 40%, and 60% incomplete). With excessive amounts of missing data (80% incomplete), CFs failed in 19% of simulations in sample A and in 16.8% in sample B. Failure occurred because, after introducing missing values, none of the analyzed specimens met the predefined ≥ 75% completeness threshold.

### Initial sample sizes of 100 specimens

**Fig. 3:**
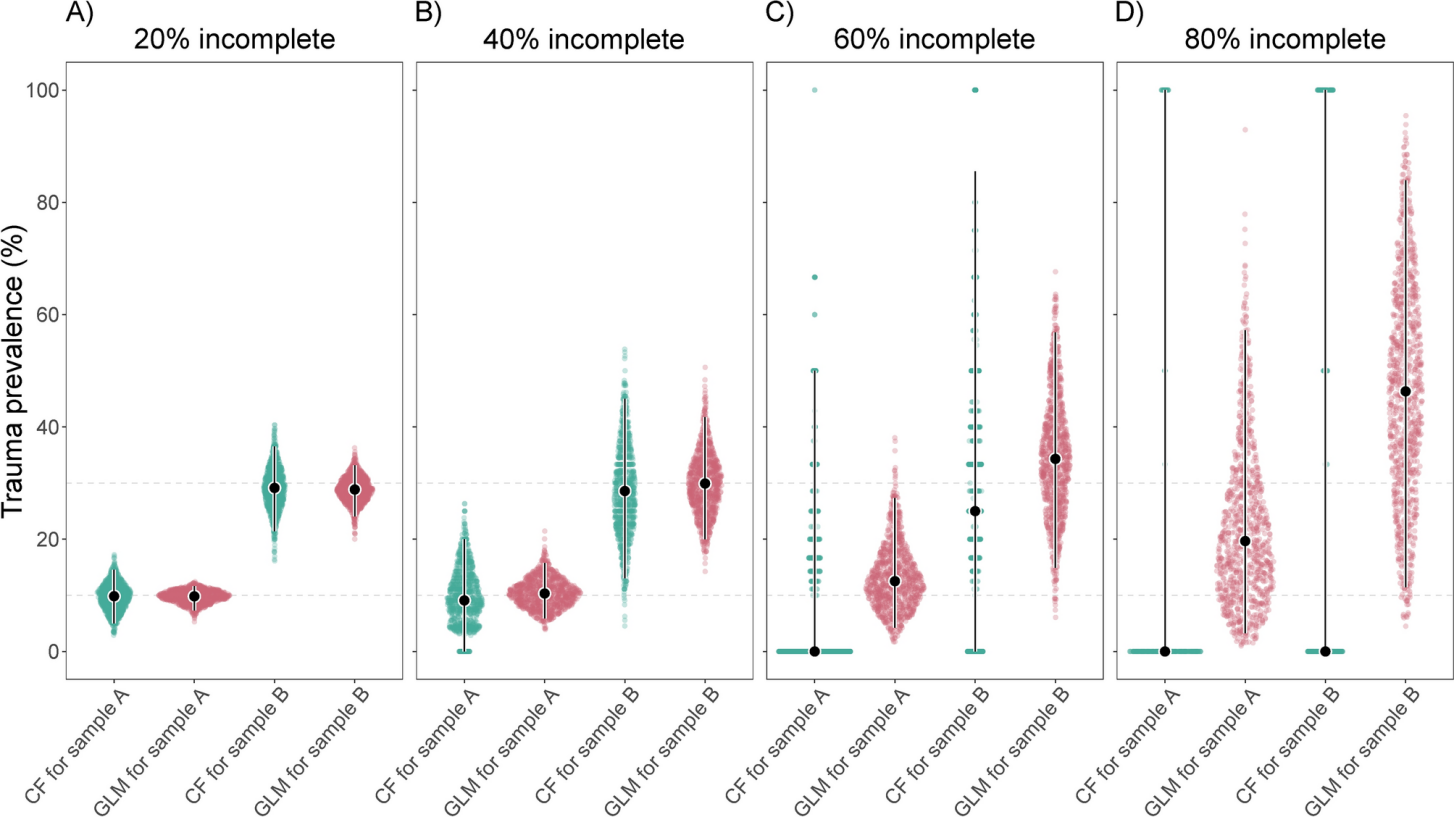
Sina plots showing the performance of CFs and GLMs in estimating cranial trauma prevalence for samples A and B (initially each *n* = 100) with increasing levels of data missingness, assessed with 1000 simulated datasets each: (**A**) 20% missing data; (**B**) 40% missing data; (**C**) 60% missing data; (**D**) 80% missing data. Color-coding denotes CF- (green) and GLM-derived (red) estimates. Dotted horizontal lines illustrate the prevalence benchmarks for samples A and B (10% and 30%, respectively). Error bars are superimposed on the distributions to display medians (dots; indicating accuracy in relation to the benchmarks) and 95% interpercentile ranges (bars; indicating precision).

To assess the effect of overall smaller sample sizes, we performed our simulation analysis again using initial sample sizes of 100 instead of 500 specimens. The generated estimates are visualized in Fig. 3, with corresponding values presented in Table 3a. Again, we observe that, with low to moderate amounts of missing data (20% and 40% incomplete), both CFs and GLMs produced similarly accurate estimates for both samples. However, with high to excessive amounts of missing data (60% and 80% incomplete), CFs tended to increasingly underestimate the benchmarks for both samples, while GLMs tended to overestimate them. As before, GLM estimates were consistently more precise than CF estimates across all incompleteness scenarios and for both samples, although precision was lower overall given the now smaller sample sizes. The difference in precision between GLMs and CFs was again most pronounced in the 80% missing data scenario, where CF-based prevalence estimates spanned the entire range from 0 to 100% in both samples A and B. CF-based estimates increasingly skewed towards 0% as the amount of missing data increased, starting at 40% incomplete, especially for sample A. For example, in the 80% missing data scenario, the number of CFs yielding a trauma prevalence estimate of 0% increased to 87.3% for sample A and 63.5% for sample B, even though the simulated raw data with missing values again never exhibited a prevalence of 0%. In contrast, GLMs always produced prevalence estimates greater than 0%, in compliance with the simulated raw data.

**Table 3:** Summary statistics on the performance of CFs and GLMs in estimating cranial trauma prevalence for samples A and B (benchmarks: 10% and 30%, respectively) with increasing levels of data missingness (on average 20%, 40%, 60%, and 80% incomplete), each of the four incompleteness scenarios assessed with 1000 simulated datasets. Initial sample sizes for samples A and B are 100.

(a) Median (and 95% interpercentile range) of trauma prevalence estimates				
Scenario	CF		GLM	
	Sample A	Sample B	Sample A	Sample B
20%	9.836 (5.000–14.516)	29.129 (21.426–36.508)	9.820 (7.315–11.648)	28.850 (24.061–33.148)
40%	9.091 (0.000–20.000)	28.571 (13.043–45.000)	10.328 (5.904–15.760)	29.913 (19.997–41.732)
60%	0.000 (0.000–50.000)	25.000 (0.000–85.500)	12.521 (4.264–27.304)	34.298 (14.936–56.832)
80%	0.000 (0.000–100.000)	0.000 (0.000–100.000)	19.665 (3.237–57.237)	46.331 (11.459–83.908)

(b) Percentage of maintaining the correct relative prevalence pattern between samples A and B		
Scenario	CF	GLM
20%	100%	100%
40%	96.7%	100%
60%	61.5%	100%
80%	2.8%	100%

(c) Percentage of producing a trauma prevalence estimate for samples A and B				
Scenario	CF		GLM	
	Sample A	Sample B	Sample A	Sample B
20%	100%	100%	100%	100%
40%	100%	100%	100%	100%
60%	98.5%	99%	100%	100%
80%	27.6%	28.2%	100%	100%

As before, GLMs always maintained the correct relative trauma prevalence pattern between samples (with A < B) across all levels of incompleteness (Table 3b). CFs only maintained the correct relative trauma prevalence pattern when the amount of missing data was low (20% incomplete). With moderate to excessive amounts of missing data (40%, 60%, and 80% incomplete), however, CFs yielded the correct relative pattern in only 96.7%, 61.5%, and 2.8% of simulations, respectively, and otherwise produced the incorrect, reverse pattern with A > B. An examination of the simulated raw datasets again confirmed that they always preserved the A < B pattern, regardless of the amounts of missing values. While GLM estimates consistently reflected this pattern based on the available raw data, CF estimates increasingly failed to do so due to the excessive exclusion of specimens that were less than 75% complete.

As also observed before, GLMs always produced trauma prevalence estimates for samples A and B across all incompleteness scenarios (Table 3c). CFs, on the other hand, only consistently produced trauma prevalence estimates when the amount of missing data ranged from low to moderate (20% and 40% incomplete). With high to excessive amounts of missing data (60% and 80% incomplete), CFs failed in 1.5% and 72.4% of simulations in sample A, and in 1% and 71.8% of simulations in sample B, respectively. Failure occurred because no specimens met the predefined ≥ 75% completeness threshold after missing values were introduced.

## Discussion

Estimating trauma prevalence from incomplete skeletal remains is hampered by the state of their completeness, as traumatic lesions that were once present may be absent and thus go undiagnosed^11–13^. This bias can result in false negative findings, increasing proportionally with decreasing completeness^11,12,14–16^, and can impede comparability between samples of varying completeness if not accounted for. Consequently, assessments of trauma prevalence based on CFs often restrict analyses to specimens meeting a minimum preservation criterion, such as ≥ 75% completeness^11,12,14,15,19,24,26–31^. While the systematic exclusion of partial remains below a predefined completeness threshold can help mitigate completeness bias, selecting an appropriate threshold remains challenging and may vary from study to study, as it involves a trade-off balancing reliable CFs against maintaining an adequate sample size, making it a potential source of bias itself. This underscores the need to integrate alternative data analysis routines, which can provide more generalized avenues for small and fragmented datasets commonly encountered in bioarchaeological investigations. GLMs are a promising solution to this problem as they can overcome the aforementioned trade-off by including even the smallest skeletal elements without requiring the prior exclusion of remains. The use of GLMs and related approaches for studying patterns of skeletal lesions has become increasingly popular among bioanthropologists in the last few decades, particularly to study entheseal changes and degenerative joint disease^34,83–88^. Recently, GLMs and their extensions have also gained traction for investigating trauma patterns^20,36,37,89–92^.

Here, we assessed how closely CF- and GLM-based trauma prevalence estimates align with the known prevalence of two once-complete cranial samples after introducing increasing amounts of missing data. This was done to explore the extent to which trauma rates in incomplete samples are adequately reflected by these two approaches. As expected, we found that accuracy and precision decreased for both CFs and GLMs as skeletal completeness decreased. This pattern was observed in both larger and smaller sample sizes but was more pronounced in the latter. When the amount of missing data was low to moderate (20% and 40% incomplete), both CFs and GLMs generated similarly accurate trauma prevalence estimates for both samples. However, with high to excessive amounts of missing data (60% and 80% incomplete), CFs tended to increasingly underestimate the benchmarks for both samples, while GLMs tended to overestimate them. Importantly, we found that GLM estimates were consistently more precise than CF estimates across all incompleteness scenarios and for both sample sizes. Alarmingly, as the amount of missing data increased, CF-based estimates skewed towards 0%, frequently yielded an incorrect reversed relative prevalence pattern across samples (with A > B), and occasionally failed to produce any prevalence estimates, particularly with small sample sizes. The shortcomings of the CF approach result from the considerable reduction of sample sizes when a completeness threshold, such as ≥ 75% as employed in this study, is applied to increasingly incomplete skeletal samples. In these cases, resulting CF estimates are based on unrepresentative subsamples comprising only a few, or sometimes no, specimens that meet the completeness threshold from the original larger sample. Therefore, we caution against using CFs in bioarchaeological contexts where samples primarily comprise small fractions of skeletal specimens.

Although GLMs consistently outperformed CFs in terms of precision, the accuracy and precision of both approaches decreased as skeletal completeness decreased, with their absolute estimates often deviating substantially from the benchmarks when missing values were excessive. This underscores the most crucial prerequisite for obtaining reliable trauma prevalence estimates that applies to both approaches, namely, striving for well-designed studies with sufficiently large and representative samples. In bioarchaeological studies, however, sample size and representativity are usually dictated by the skeletal remains preserved and available from excavations, rather than by study design. In our view, it is therefore indispensable for any trauma prevalence study to quantify the completeness of all available skeletal remains in a sample prior to analysis to determine its representativity, which impacts the choice of analytical approach and the reliability and interpretation of results.

Even though our methodological exploration included simulated datasets with excessive amounts of missing data (80% incomplete) to exemplify preservation-related effects on trauma prevalence estimates, we generally consider such incomplete samples unsuitable for studying trauma rates, regardless of the approach used. In instances where critically incomplete samples are the only option available for analysis, it becomes even more crucial to quantify their completeness, report their state of preservation, and be cautious regarding the limits of interpretations. In this context, it is important to note that while CFs provide only a single point estimate of trauma prevalence, GLM-derived trauma prevalence predictions include two estimates: a point estimate of mean prevalence and a compatibility interval of the error around the mean, signaling uncertainty in the estimate, such as resulting from the underlying sample size. To ensure comparability among approaches in our simulation framework, we relied only on CF and GLM point estimates, without further considering the GLM compatibility intervals. In bioarchaeological applications, however, the GLM compatibility intervals can help evaluate the estimate and its uncertainty, potentially resulting from limited sample sizes—a feature absent in the CF approach.

In the 60% and 80% missing data scenarios, we observe that GLM estimates not only range widely around the benchmarks but also tend to overestimate trauma prevalence (Figs. 2C, D and 3C, D). By default, GLMs allow for the explicit specification of the completeness score for which trauma prevalence estimates are to be predicted. To ensure comparability in our simulation framework between the GLM and CF approaches, the latter employing a ≥ 75% completeness threshold, we predicted GLM trauma prevalence across the entire 75% to 100% completeness score range and averaged those estimates. We suspect that an overestimation of trauma prevalence might result from an underrepresentation of largely complete specimens in the 60% and especially in the 80% incomplete datasets, limiting the models’ references for predicting prevalence among complete specimens, which appears to lead to an over-extrapolation from lower completeness scores. Indeed, when we predicted for the sequence of 50%-75% completeness instead, estimates were lower overall, while relative prevalence patterns across samples remained constant (results not shown). Therefore, in cases of largely incomplete samples, we advise not to take the absolute GLM trauma predictions at face value but to focus on relative prevalence patterns between samples instead. This conclusion holds implications for previous studies that utilized such model-based predictions to infer cranial trauma prevalence from incomplete fossil remains of Late Pleistocene humans^36,37^. It cautions against overinterpreting predicted absolute trauma prevalence values, while reinforcing findings on relative trauma patterns in Neanderthals and Upper Paleolithic humans, including similar overall and sex-specific cranial trauma rates in the two taxa along with possible age-related differences^36,37^.

Another conceptual advantage of GLMs over CFs—not integrated into our study for the sake of direct comparability of the approaches—is their ability to simultaneously explore the effects of multiple predictor variables and their interactions on trauma prevalence (such as age at death, sex, or archaeological period). This stands in contrast to CFs, which can only assess a single predictor variable at a time. Moreover, in the form of generalized linear mixed models (hereafter, GLMMs), their utility is further enhanced by the ability to account for repeated observations and further dependency structures of the data through partial pooling using random effects^35,93,94^. This can help to avoid potential pseudoreplication, an issue that can arise in bioarchaeological trauma studies when linear models are implemented without specifying appropriate random effects—such as in cases where multiple cranial elements affected by the same fracture, or multiple traumas on the same individual, are recorded separately. The versatility of GL(M)Ms comes at the cost of higher computational complexity compared to CFs. However, a recently published step-by-step workflow in the programming language R, which requires minimal coding skills and is directed at users of different experience levels^35^, greatly facilitates the implementation of GL(M)Ms, alongside other best-practice and troubleshooting literature^93–100^.

In conclusion, we generally recommend GL(M)Ms for estimating trauma prevalence from incomplete skeletal samples and caution against using CFs, especially when dealing with largely incomplete and small samples. In addition to their overall superior performance demonstrated in this study, GL(M)Ms offer conceptual advantages that are typically pertinent to most bioarchaeological investigations. These include the ability to accommodate complex data structures, such as hierarchical designs, and to assess multiple predictor variables simultaneously in both the estimation of trauma prevalence and their statistical comparison between samples. We demonstrated that neither approach is fully reliable when dealing with largely incomplete samples. Nevertheless, in such cases, GL(M)Ms can at least help to assess relative prevalence patterns between samples. In this context, it is important to note that, while powerful, GL(M)Ms are only statistical models and should thus be used with caution. In particular, if the goal is causal inference, their formulation should be based on appropriate causal models^101^.

Lastly, it is important to emphasize that our simulation study is grounded on empirical data relevant to archaeological contexts of the Last Glacial, incorporating real-life cases of blunt force cranial trauma which are realistic for hunter-gatherers (resulting from interpersonal violence, animal encounters, falls from a height, and projectile trauma from arrow shots) and information about the completeness of cranial remains from the Upper Paleolithic period. Consequently, our findings may not necessarily apply to other archaeological contexts, such as medieval or early modern periods, where markedly different injury scenarios may have existed (including sharp force trauma, explosives, and gunshot trauma), and to taphonomic contexts where the relative representation patterns of skeletal remains might be different. Moreover, our study was restricted to cranial remains and focused exclusively on the impact of data missingness and sample sizes on trauma prevalence estimates using the two approaches of CFs and GLMs. Future research could, for example, expand such methodological explorations of sample incompleteness on trauma prevalence estimates to the post-cranial skeleton, explore more complex research designs involving multiple predictor variables and their interactions, and compare the accuracy of different GL(M)M algorithms and software packages for predicting trauma prevalence.

## Supplementary Information

Supplementary Information 1.

Supplementary Information 2.

## Data Availability

All data and code needed for reproducing or extending our analyses are presented or referenced in the main text and supplementary materials.
