# Supplementary material for: Estimating trauma prevalence from incomplete human skeletal remains
Source: Sci Rep. 2024 Nov 12;14:27713. doi: 10.1038/s41598-024-76231-1 (PMC11557604; doi:10.1038/s41598-024-76231-1)
Supplement: Supplementary file 2 — Supplementary Information 2. [file 41598_2024_76231_MOESM2_ESM.docx]

**Supplementary Information**

**Estimating trauma prevalence from incomplete human skeletal remains**

Judith Beier, Matteo Santon, Hannes Rathmann


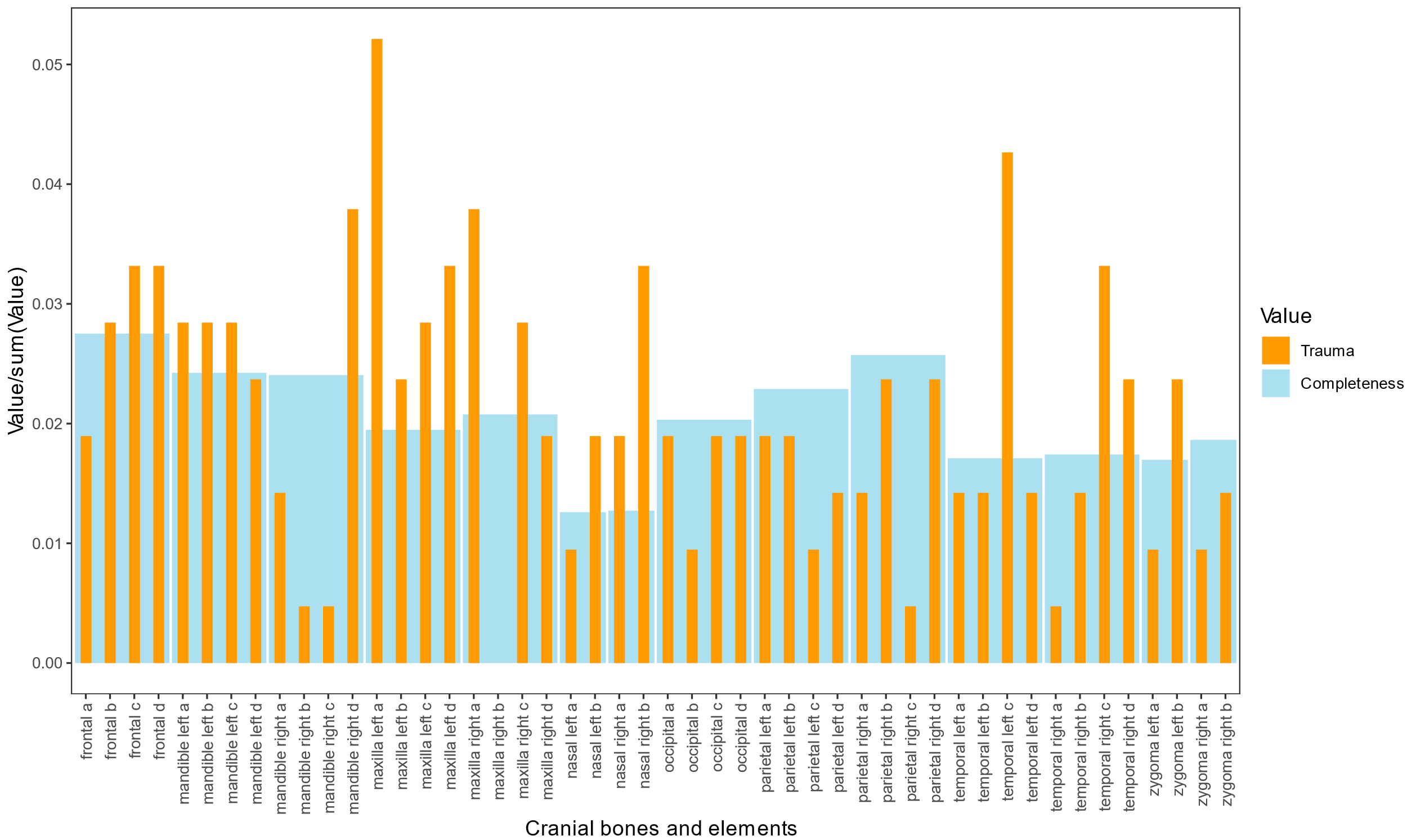


***Figure S1.*** *Bar plot visualizing the empirical data on real-life cranial trauma patterns and differential archaeological bone representation underlying our data simulations. The plot shows the 14 major cranial bones, each further divided into individual elements, and (in orange) the number of individual elements affected by trauma within the pool of 40 published forensic and clinical trauma cases with injury scenarios realistic for prehistoric hunter-gatherer populations, and (in blue) the effective sample sizes as a measure for the relative over- and underrepresentation of the 14 cranial bones in a published Upper Paleolithic cranial sample. See methods section in the main text for details and references. Both the numbers of elements with trauma and the effective sample sizes are normalized [Value/sum(Value)] for comparability. The plot illustrates some divergence between the bones that are more or less frequently affected by trauma in real-life cases and those that are more or less commonly represented in the archaeological record. For instance, some bones, like the right mandible, are rarely affected by trauma yet are relatively well-represented in the archaeological record, while others, such as the nasal bones and left maxilla, are often affected by trauma but less frequently preserved. By incorporating these empirical datasets, we accounted for this variability in our simulations.*
